# *Toxoplasma gondii* infection in white spoonbills (*Platalea leucorodia*) from Henan Province, China

**DOI:** 10.1080/22221751.2020.1854057

**Published:** 2020-12-10

**Authors:** Yurong Yang, Nan Jiang, Shilin Xin, Longxian Zhang

**Affiliations:** College of Veterinary Medicine, Henan Agricultural University, Zhengzhou, People’s Republic of China

**Keywords:** *T. gondii*, *Platalea leucorodia*, isolation, genotype, virulence

## Abstract

*Toxoplasma gondii* oocysts in the environment are a threat to humans and animals. This study is aimed to evaluate the prevalence of *T. gondii* in white spoonbills and isolate viable *T. gondii* from white spoonbills. In 28.6% (2/7) of white spoonbills, *T. gondii* antibodies were found in heart juice by the modified agglutination test (cut-off: 1:4). *T. gondii* DNA was detected in tissues of 42.9% (3/7) white spoonbills. One viable *T. gondii* strain, named TgSpoonbillCHn1, was isolated from the myocardium of a white spoonbill by bioassay in mice. DNA extracted from TgSpoonbillCHn1 tachyzoites was characterized by PCR-restriction fragment length polymorphism with ten markers and the virulence genes ROP5 and ROP18. The results revealed that it was ToxoDB#2 (Type III). The ROP18/ROP5 genotype combination predicts that this strain is avirulent for mice, which is supported by the infection experiments in mice. This is the first report of the isolation of viable *T. gondii* strain from white spoonbil. The prevalence of *T. gondii* in white spoonbills may be indicative of environmental contamination of oocysts. This report provides direct evidence of white spoonbill as an intermediate host of *T. gondii*.

**Dear editor:** *Toxoplasma gondii* is an obligate intracellular parasite that has a worldwide distribution and can infect almost all warm-blood animals, including birds [1]. However, *T. gondii* behaves differently in different hosts. Birds are considered resistant to clinical toxoplasmosis. The serological response for *T. gondii* infection in birds was erratic and transient, the serological results of *T. gondii* should be interpreted with caution [2,3]. White spoonbill (*Platalea leucorodia*), as a large wading bird, they obtain food from water, sludge, or the ground. Their food includes small aquatic creatures, insects, crustaceans, tiny fish, and loach. The infection status of *T. gondii* in white spoonbill is an excellent indicator to monitor the environment for *T. gondii* oocysts contamination. However, there is still a lack of direct evidence that *T. gondii* can infect spoonbill.

In this study, from September 2018 to June 2020, eleven adult white spoonbills died of bloody mass stools in a zoo (34°46′ N, 113°39′ E, Henan, China) (Table 1, Figure S1). These birds were fed fish and loaches, also foraged from the artificial lake. Fresh tissue samples from seven white spoonbills were collected. These samples were submitted to the Laboratory of Veterinary Pathology of Henan Agricultural University (Zhengzhou, China) for pathological diagnosis and also allow us to investigate *T. gondii* infection in white spoonbills.

**Table 1.** Background data and isolation of *Toxoplasma gondii* from white spoonbills (*Platalea leucorodia*).

Samples ID	Received date	Sex, age	Clinical signs	Pathological findings	MAT titers^a^	PCR^b^	Mice bioassay ^c^
Case#1	Sep 27, 2018	Female adult	Loose stools with fresh blood	nd	nd	nd	nd
Case#2	Sep 30, 2018	Female adult	Loose stools with fresh blood	nd	nd	nd	nd
Case#3	Oct 3, 2018	Female adult	Salivate, depressed, anorexia	Hydropericardium, liver congestion	<1:2	+	0/4
Case#4	Oct 3, 2018	Female adult	Depressed, anorexia	Myocardial hemorrhage, glandular stomach mucosal hemorrhage, liver congestion	1:16	+	1/5, 4/5, 5/5, 5/5, 2/2, 3/3, 3/3, 5/5
							
Case#5	Oct 5, 2018	Female adult	Depressed, anorexia	Myocardial hemorrhage	nd	nd	nd
Case#6	Oct 5, 2018	Female adult	Salivate, depressed, anorexia	Myocardial hemorrhage	nd	nd	nd
Case#7	Oct 7, 2018	Male, adult	Salivate, loose and black stools, depressed, anorexia	Hydropericardium, endocardial hemorrhage	1:16	-	nd
Case#8	Oct 7, 2018	Female adult	Salivate, depressed, anorexia	Splenomegaly, liver congestion, kidney hemorrhage	<1:2	-	nd
Case#9	Oct 11, 2018	Female adult	Loose stools with fresh blood, salivate, depressed, anorexia	Pulmonary congestion, kidney hemorrhage	<1:2	-	nd
Case#10	Nov 11, 2018	Female adult	Loose and black stools, depressed, anorexia	Enlarged liver and kidney	<1:2	-	nd
Case#11	Sep 13, 2019	Female adult	Loose and black stools, depressed, anorexia	Heart coronary fat hemorrhage, liver congestion	<1:2	+	nd

^a^ Titration: 1:2–1:8192.

^b^ Primer were TOX5/TOX8.

^c^ No. of positive mice/No. of inoculated mice.

nd: not done.

The degree of fat storage and muscle development in these white spoonbills were well. Bloody stools (6/11), salivate (4/11), hydropericardium (2/9), myocardium or heart coronary fat hemorrhage (5/9), white pulp necrosis (4/7) were observed by gross and microscopic examination (Figure S1 B–E). Pathological diagnosis showed that adenovirus infection was the major cause of death in these white spoonbills. *T. gondii* parasites were not found in tissue sections of all seven white spoonbills.

*T. gondii* antibodies were identified by the modified agglutination test (MAT) (cut off = 1:4) [4]. Our survey indicated that 28.6% (2/7, case#4 and case#7) (95% CI, 7.56%–64.76%) of the white spoonbills were seropositive for *T. gondii*, with titres of 1:16 (Table 1). The current understanding of the specificity and sensitivity of the serological diagnosis of *T. gondii* infection in birds is limited. Viable *T. gondii* were isolated from 6 of 1025 chickens with MAT titre of <1:5 by bioassay in cats [5]. Cabezón reported 6.2% *T. gondii* antibodies in the Eurasian spoonbill (5/81) by MAT, with titres greater than or equal to 1:25 [6]. Work found *T. gondii* infection in one species of *Pelecaniformes*, a red-footed booby (*Sula sula*), and parasites were confirmed by immunohistochemical (IHC) staining in the heart and cerebrum [7]. However, the serum antibody reaction to *T. gondii* was not reported.

PCR assays for *T. gondii* were performed using primer Tox-8 and Tox-5 [8]. *T. gondii* DNA was detected in tissues of 42.9% (3/7) (95% CI, 15.75%–75.02%) white spoonbills. *T. gondii* DNA was detected in myocardium digestive juices of white spoonbills (case#3, case#4), and in lung and heart of white spoonbill case#11.

The myocardium (20 g) of white spoonbill case#3 and case#4 was bioassayed in mice individually [1]. For the TOX#25-4 group (case#4), mouse (M#796) had seroconversion antibodies for *T. gondii*, and cysts (*n* = 120 in the whole brain) were found in the brain at 43 DPI (Figure S1 F). *T. gondii* cysts were verified by IHC (Figure S1 G). The *T. gondii* from the mouse brain was propagated in cell culture successfully (15 DPI) and designated as TgSpoonbillCHn1. DNA samples extracted from *T. gondii* tachyzoites in cell cultures were characterized by PCR-RFLP [9,10]. Its genetic typing was ToxoDB#2 (type III). ToxoDB#2 is widely distributed worldwide [11]. ToxoDB#2 *T. gondii* strains were previously found in cats [12] and sheep [13] from central China, indicating that, except for ToxoDB#9, ToxoDB#2 is one of the major endemic genotypes in China. Until now, there were more than one hundred viable *T. gondii* strains isolated from avian species (excluding chickens) worldwide, most from *Anseriformes*, *Columbiformes* and *Accipitriformes* [2,3]. However, only one *T. gondii* strain isolated from *Pelecaniformes* [14]. Genetic diversity of *T. gondii* isolates from birds follows the global patterns, with ToxoDB #1, #3, and #2 being dominant in Africa and Europe [3]. 22 DNA extracted from tissues of birds from China were genotyped, they were ToxoDB #10, #1, #3 and #9 [3]. TgSpoonbillCHn1 was the first genotyped strain from viable *T. gondii* isolate in birds from China.

The virulence of TgSpoonbillCHn1 was evaluated in Swiss mice. The ROP18/ROP5 genotype combination (3/3) suggests this strain is avirulence for mice, which matched with the mouse virulence evaluation in this study (Table S1).

The source of *T. gondii* infection for these white spoonbills is less clear. Vertical transmission of *T. gondii* is sporadic in chicken; however, this is unclear in other species of birds, including white spoonbill. These white spoonbills were hatched and grew in a zoological park. White spoonbills may have acquired *T. gondii* through the ingestion of oocysts from felids feces or mechanical transport hosts (fish and loaches in the feed). This indicated that *T. gondii* oocysts contaminate the habitat environment (water, soil) of white spoonbills. The high seropositive of *T. gondii* antibodies in captive tigers (80%) from central China support this hypothesis [15].

This is the first report on the isolation of *T. gondii* from white spoonbill, providing direct evidence that white spoonbill is an intermediate host of *T. gondii*. White spoonbills could serve as a good sentinel animal for *T. gondii* contamination in the environment.

## Supplementary Material

fig_1_300dpi.tif

Fig_S1.docx

Table_S1.docx

## References

[1] Dubey JP. Toxoplasmosis of animals and humans. Boca Raton (FL): CRC Press, Taylor & Francis Group; 2010, p. 1–313.

[2] Dubey JP. A review of toxoplasmosis in wild birds. Vet Parasitol. 2002;106:121–153. 12031816 10.1016/s0304-4017(02)00034-1

[3] Dubey JP, Murata FHA, Cerqueira-Cézar CK, et al. Epidemiologic significance of *Toxoplasma gondii* infections in turkeys, ducks, ratites, and other wild birds: 2009-2020. Parasitology. 2020:1–30. DOI:10.1017/S0031182020001961. PMC11010194 33070787

[4] Dubey JP, Desmonts G. Serological responses of equids fed *Toxoplasma gondii* oocysts. Equine Vet J. 1987;19:337–339. 3622463 10.1111/j.2042-3306.1987.tb01426.x

[5] Dubey JP, Laurin E, Kwok OCH. Validation of the modified agglutination test for detection of *Toxoplasma gondii* in free-range chickens by using cat and mouse bioassay. Parasitology. 2016;143:314–319. 26625933 10.1017/S0031182015001316

[6] Cabezón O, García-Bocanegra I, Molina-López R, et al. Seropositivity and risk factors associated with *Toxoplasma gondii* infection in wild birds from Spain. PLoS One. 2011;6(12):e29549. 22216311 10.1371/journal.pone.0029549 PMC3245288

[7] Work TM, Massey JG, Lindsay D, et al. Toxoplasmosis in three species of native and introduced Hawaiian birds. J Parasitol. 2002;88(5):1040–1042. 12435157 10.1645/0022-3395(2002)088[1040:TITSON]2.0.CO;2

[8] Reischl U, Bretagne S, Krüger D, et al. Comparison of two DNA targets for the diagnosis of toxoplasmosis by real-time PCR using fluorescence resonance energy transfer hybridization probes. BMC Infect Dis. 2003;3:7. 12729464 10.1186/1471-2334-3-7 PMC156600

[9] Su C, Shwab EK, Zhou P, et al. Moving towards an integrated approach to molecular detection and identification of *Toxoplasma gondii*. Parasitology. 2010;137:1–11. 19765337 10.1017/S0031182009991065

[10] Shwab EK, Jiang T, Pena HF, et al. The ROP18 and ROP5 gene allele types are highly predictive of virulence in mice across globally distributed strains of *Toxoplasma gondii*. Int J Parasitol. 2016;46:141–146. 26699401 10.1016/j.ijpara.2015.10.005

[11] Shwab EK, Saraf P, Zhu XQ, et al. Human impact on the diversity and virulence of the ubiquitous zoonotic parasite *Toxoplasma gondii*. P Natl Acad Sci USA. 2018;115:E6956–E6963. 10.1073/pnas.1722202115 PMC6055184 29967142

[12] Yang Y, Ying Y, Verma SK, et al. Isolation and genetic characterization of viable *Toxoplasma gondii* from tissues and feces of cats from the central region of China. Vet Parasitol. 2015;211:283–288. 26033402 10.1016/j.vetpar.2015.05.006

[13] Jiang N, Su R, Jian F, et al. *Toxoplasma gondii* in lambs of China: heart juice serology, isolation and genotyping. Int J Food Microbiol. 2020;322:108563. 32113068 10.1016/j.ijfoodmicro.2020.108563

[14] Silva MA, Pena HFJ, Soares HS, et al. Isolation and genetic characterization of *Toxoplasma gondii* from free-ranging and captive birds and mammals in Pernambuco state, Brazil. Revista Brasileira de Parasitologia Veterinária. 2018;27:481–487. 30184004 10.1590/S1984-296120180059

[15] Yang Y, Dong H, Su R, et al. Direct evidence of an extra-intestinal cycle of *Toxoplasma gondii* in tigers (*Panthera Tigris*) by isolation of viable strains. Emerg Microbes Infec. 2019;8:1550–1552. 31661400 10.1080/22221751.2019.1682471 PMC6830256

