# Supplementary figures and images for: Toxoplasma gondii infection in white spoonbills (Platalea leucorodia) from Henan Province, China
Source: Emerg Microbes Infect. 2020 Dec 10;9(1):2619–21. doi: 10.1080/22221751.2020.1854057 (PMC7733910; doi:10.1080/22221751.2020.1854057)

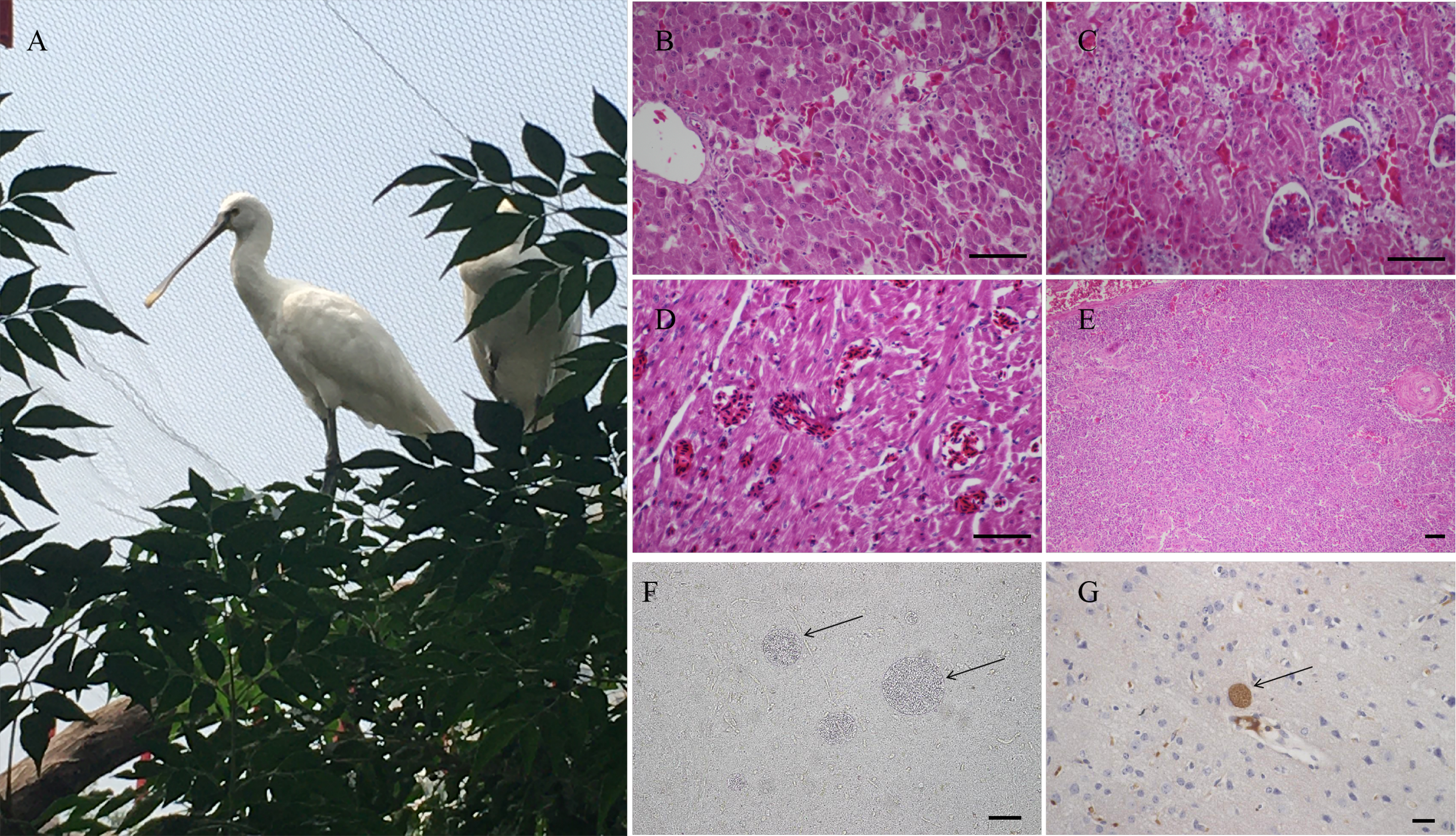

Supplement: fig_1_300dpi.tif [file TEMI_A_1854057_SM3532.tif]
