# Supplementary material for: Toxoplasma gondii infection in white spoonbills (Platalea leucorodia) from Henan Province, China
Source: Emerg Microbes Infect. 2020 Dec 10;9(1):2619–21. doi: 10.1080/22221751.2020.1854057 (PMC7733910; doi:10.1080/22221751.2020.1854057)
Supplement: Fig_S1.docx [file TEMI_A_1854057_SM3531.docx]

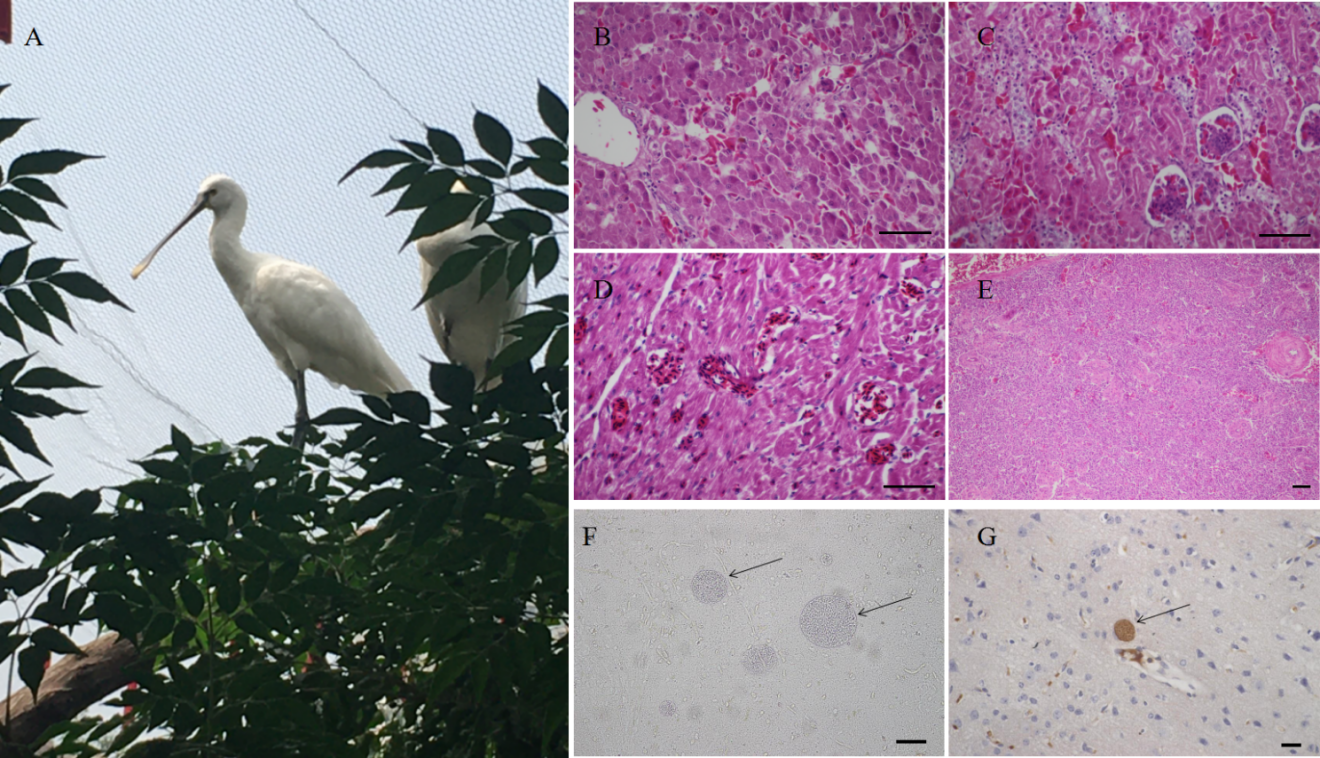


**Figure S1 Histopathological findings in the white spoonbills (*Platalea leucorodia*).**

A. White spoonbills from the zoo (China);

B. Hepatocyte necrosis, [acute liver congestion](file:///C:/Users/yyr/AppData/Local/youdao/dict/Application/7.5.2.0/resultui/dict/javascript:;), case#3, liver, white spoonbill, H&E;

C. [Kidney hemorrhage](file:///C:/Users/yyr/AppData/Local/youdao/dict/Application/7.5.2.0/resultui/dict/javascript:;), [acute glomerulonephritis](file:///C:/Users/yyr/AppData/Local/youdao/dict/Application/7.5.2.0/resultui/dict/javascript:;), case#3, kidney, white spoonbill, H&E;

D. Cardiomyocyte necrosis, congestion, case#4, white spoonbill, H&E;

E. White pulp necrosis, many reticular cell proliferation around lymphangion, case#8, spleen, white spoonbill, H&E;

F. *T. gondii* TgSpoonbillCHn1 cysts in the brain, mouse (Tox 25-4 M#796), 43 DPI, squashed section, unstained;

G. *T. gondii* TgSpoonbillCHn1 cysts in the brain, mouse (Tox 25-4 M#796), 43 DPI, IHC;

Bar = 50 μm.
