# Supplementary material for: Toxoplasma gondii infection in white spoonbills (Platalea leucorodia) from Henan Province, China
Source: Emerg Microbes Infect. 2020 Dec 10;9(1):2619–21. doi: 10.1080/22221751.2020.1854057 (PMC7733910; doi:10.1080/22221751.2020.1854057)
Supplement: Table_S1.docx [file TEMI_A_1854057_SM3530.docx]

**Table S1. Virulence of *Toxoplasma gondii* TgSpoonbillCHn1 strain in Swiss mice.**

| **No. of tachyzoites** | **No. of infection/No. of inoculation (%)** | **Mice survival days/No. of surviving mice** | **No. of brain cysts** |
| --- | --- | --- | --- |
| **10^6^** | 5/5 (100%) | 22DPI/1, ≥60DPI/4 | 157.5±47.5 |
| **10^5^** | 5/5 (100%) | ≥ 60DPI/5 | 268.0±95.9 |
| **10^4^** | 5/5 (100%) | 21DPI/1, ≥ 60DPI/4 | 148.0±102.9 |
| **10^3^** | 4/5 (80%) | ≥ 60DPI/5 | 12.5±4.8 |
| **10^2^** | 4/5 (80%) | ≥ 60DPI/5 | 97.5±33.5 |
| **10^1^** | 3/5 (60%) | ≥ 60DPI/5 | 20.0±10.0 |
| **1** | 1/5 (20%) | ≥ 60DPI/5 | 240.0 |
| **<1** | 0/5 (-) | ≥ 60DPI/5 | Not found |
| **Blank control** | 0 | ≥ 60DPI/5 | Not found |
